# Heterogeneous Nuclear Ribonucleoprotein K Is Involved in the Estrogen-Signaling Pathway in Breast Cancer

**DOI:** 10.3390/ijms22052581

**Published:** 2021-03-04

**Authors:** Erina Iwabuchi, Yasuhiro Miki, Takashi Suzuki, Hisashi Hirakawa, Takanori Ishida, Hironobu Sasano

**Affiliations:** 1 Department of Pathology, Tohoku University Graduate School of Medicine, Sendai 980-8575, Japan; e-iwabuchi@med.tohoku.ac.jp; 2 Department of Disaster Obstetrics and Gynecology, International Research Institute of Disaster Science (IRIDes), Tohoku University, Sendai 980-8575, Japan; miki@patholo2.med.tohoku.ac.jp; 3 Department of Pathology and Histotechnology, Tohoku University Graduate School of Medicine, Sendai 980-8575, Japan; t-suzuki@patholo2.med.tohoku.ac.jp; 4 Department of Surgery, Tohoku Kosai Hospital, Sendai 980-0803, Japan; hirakawa@tohokukosai.com; 5 Department of Breast and Endocrine Surgical Oncology, Tohoku University Graduate School of Medicine, Sendai 980-8575, Japan; takanori@med.tohoku.ac.jp

**Keywords:** hnRNPK, breast cancer, protein-protein interaction, estrogen receptor

## Abstract

Heterogeneous nuclear ribonucleoprotein K (hnRNPK) transcripts are abundant in estrogen receptor (ER)- or progesterone receptor (PR)-positive breast cancer. However, the biological functions of hnRNPK in the ER-mediated signaling pathway have remained largely unknown. Therefore, this study analyzes the functions of hnRNPK expression in the ER-mediated signaling pathway in breast cancer. We initially evaluated hnRNPK expression upon treatment with estradiol (E2) and ICI 182,780 in the ERα-positive breast carcinoma cell line MCF-7. The results revealed that E2 increased hnRNPK; however, hnRNPK expression was decreased with ICI 182,780 treatment, indicating estrogen dependency. We further evaluated the effects of hnRNPK knockdown in the ER-mediated signaling pathway in MCF-7 cells using small interfering RNAs. The results revealed that hnRNPK knockdown decreased ERα expression and ERα target gene pS2 by E2 treatment. As hnRNPK interacts with several other proteins, we explored the interaction between hnRNPK and ERα, which was demonstrated using immunoprecipitation and proximity ligation assay. Subsequently, we immunolocalized hnRNPK in patients with breast cancer, which revealed that hnRNPK immunoreactivity was significantly higher in ERα-positive carcinoma cells and significantly lower in Ki67-positive or proliferative carcinoma cells. These results indicated that hnRNPK directly interacted with ERα and was involved in the ER-mediated signaling pathway in breast carcinoma. Furthermore, hnRNPK expression could be an additional target of endocrine therapy in patients with ERα-positive breast cancer.

## 1. Introduction

Breast cancer is one of the most common cancers among women. Estrogen, the primary female sex steroid hormone, and the estrogen receptor (ER) signaling pathway contribute to the progression of breast cancer [1]. Tamoxifen, a selective ER modulator (SERM) that binds to the ER and antagonizes the effects of estrogen, has been the mainstay of endocrine therapy in patients with breast cancer, especially premenopausal ones [2]. However, primary or acquired resistance to tamoxifen is clinically unavoidable, and the mechanisms underlying this resistance have remained largely unknown [3].

Heterogeneous nuclear ribonucleoprotein K (hnRNPK) has been detected in the nucleus, cytoplasm, and mitochondria of cells and is involved in chromatin remodeling, transcription, splicing, and translation processes [4]. Furthermore, hnRNPK is overexpressed in the nuclei and cytoplasm of several types of cancer cells, including head-and-neck/oral squamous cell carcinomas (SCCA), and its aberrant cytoplasmic localization is associated with a poor prognosis, suggesting its involvement in cancer progression [5,6]. In another study, hnRNPK is mainly expressed in the nucleus and is associated with a poor prognosis in patients with urinary bladder cancer [7]. In addition, another study has found that both nuclear and cytoplasmic hnRNPK significantly increased in patients with colorectal cancer with Dukes’ C staging, which led to adverse clinical outcomes in these patients and to those whose tumors had a low or negative nuclear hnRNPK score [8]. Moreover, hnRNPK inhibits cell proliferation in gastric carcinoma cells [9]. In breast cancer, patients with histological grade III cancer had more hnRNPK proteins than those with lower grades according to an analysis using Western blotting [10]. In addition, hnRNPK transcripts are more abundant in ER- or progesterone receptor (PR)-positive breast cancer or luminal-type cancer [11]. The hnRNPK protein has recruited diverse molecular partners and could act as a docking platform involved in such processes as transcription, RNA processing, and translation [12,13]. For instance, long non-coding RNA facilitates hnRNPK-mediated stability and transactivation of β-catenin in neuroblastoma cells [14]. Therefore, the possible interaction between hnRNPK and ER is reasonably postulated to be involved in the ER-mediated signaling pathway of breast cancer. However, the biological functions of hnRNPK in the ER-mediated signaling pathway in breast cancer have remained virtually unexplored. In this study, we analyzed the function of hnRNPK as a binding protein in the ER-mediated signaling pathway in breast cancer.

## 2. Results

### 2.1. The Effects of Estradiol (E2) in MCF-7 Cells

**Figure 1:**
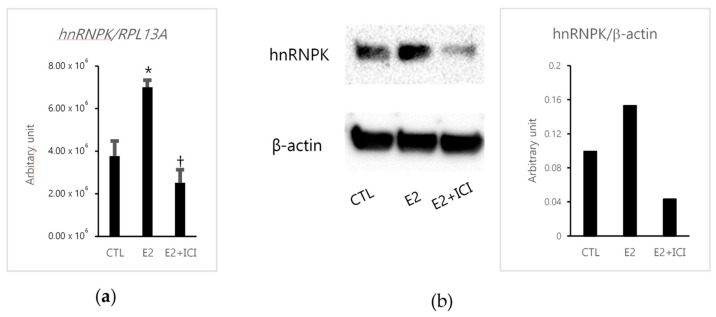
The effects of estradiol (E2) in MCF-7 cells. (**a**) Real-time PCR analysis of heterogeneous nuclear ribonucleoprotein K (hnRNPK). MCF-7 cells were treated with E2 alone or E2 combined with ICI 182,780 (E2+ICI) for 48 h and before RT–PCR. * *p* = 0.0003 versus control (CTL) for E2; † *p* = 0.0359 versus CTL for E2+ICI. (**b**) Western blotting assay of hnRNPK (65 kDa). MCF-7 cells were treated with E2 alone or E2+ICI for 72 h. β-actin (40 kDa) was used as the loading control. The full-length Western blot images are summarized in Supplementary Figure S1a.

First, we studied the expression of hnRNPK using estradiol (E2) and ERα antagonist ICI 182,780 treatment using the ERα-positive breast carcinoma cell line MCF-7 to explore the potential association between hnRNPK and the ER-mediated signaling pathway. E2 significantly increased the hnRNPK expression levels compared with the control (Figure 1). However, hnRNPK expression levels significantly decreased by the combined treatment of E2 and ICI 182,780 (Figure 1).

### 2.2. The Effects of E2 in hnRNPK-Knockdown-MCF-7 Cells

**Figure 2:**
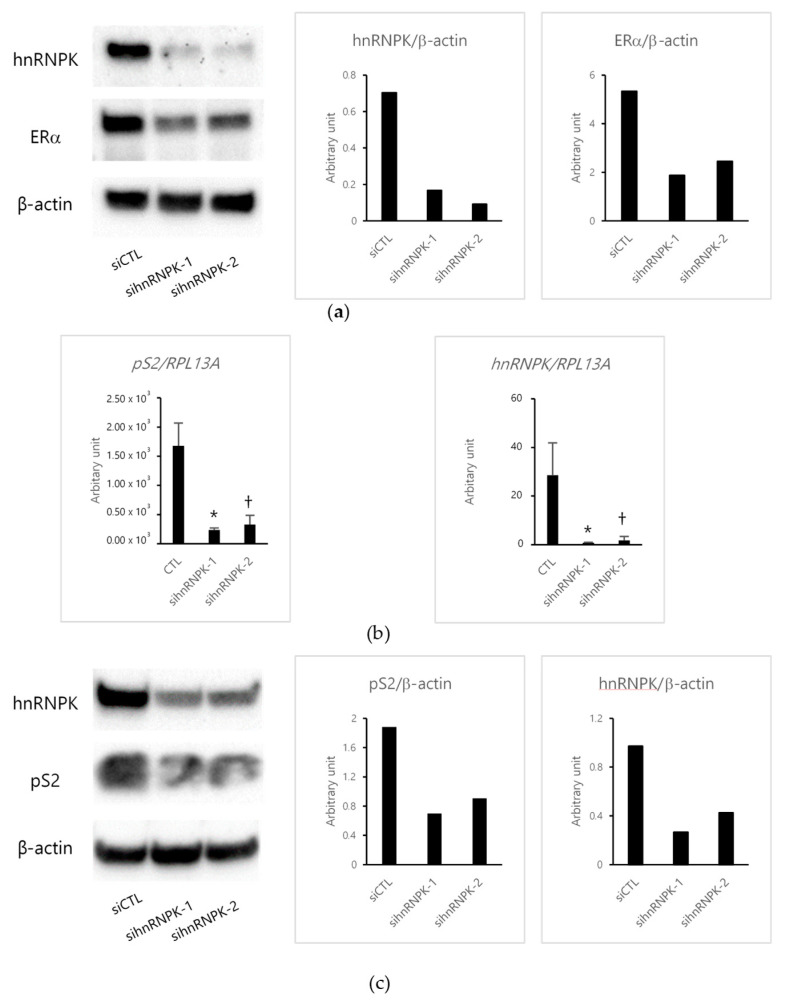
The effects of estradiol (E2) in hnRNPK-knockdown MCF-7 cells. (**a**) Western blotting assay of hnRNPK (65 kDa) and estrogen receptor (ER) α (66 kDa) in MCF-7 cells transfected with hnRNPK-specific small interfering RNAs (siRNAs) (sihnRNPK-1 and sihnRNPK-2) or negative control siRNA (siCTL) for 72 h. β-actin was the loading control. (**b**) Real-time PCR analysis of pS2 and hnRNPK in MCF-7 cells transfected with sihnRNPK-1 and sihnRNPK-2 or siCTL and then treated with E2 for 24 h. Left panel, * *p* = 0.0007 versus siCTL for sihnRNPK-1; † *p* = 0.0010 versus siCTL for sihnRNPK-2. Right panel, * *p* = 0.0080 versus siCTL for sihnRNPK-1; † *p* = 0.0094 versus siCTL for sihnRNPK-2. (**c**) Western blotting assay of pS2 (13 kDa) and hnRNPK (65 kDa) in MCF-7 cells transfected with sihnRNPK-1 and sihnRNPK-2 or siCTL and then treated with E2 for 48 h. β-actin was the loading control. The full-length Western blot images are illustrated in Figure S1b,c.

Then, we investigated the effects of E2 using small interfering RNAs (siRNAs). The expression of hnRNPK was suppressed (from 86.8% to 76.1%) in MCF-7 cells transfected with either of two hnRNPK-specific siRNAs (sihnRNPK-1 and sihnRNPK-2), but not in those transfected with negative control siRNA (siCTL) (Figure 2a). In addition, we confirmed the effects of ERα on cells transfected with hnRNPK-specific siRNAs. ERα expression was also suppressed (from 85.2% to 54.4%) in hnRNPK-knockdown MCF-7 cells compared with the control (Figure 2a). Then, we examined the effects of E2 treatment on ERα target gene pS2 expressions in cells transfected with hnRNPK-specific siRNAs. The depletion of hnRNPK significantly decreased pS2 expression (Figure 2b,c). In addition, hnRNPK expression induced by E2 was suppressed in hnRNPK-knockdown MCF-7 cells (Figure 2b,c).

### 2.3. hnRNPK and ERα Interaction

**Figure 3:**
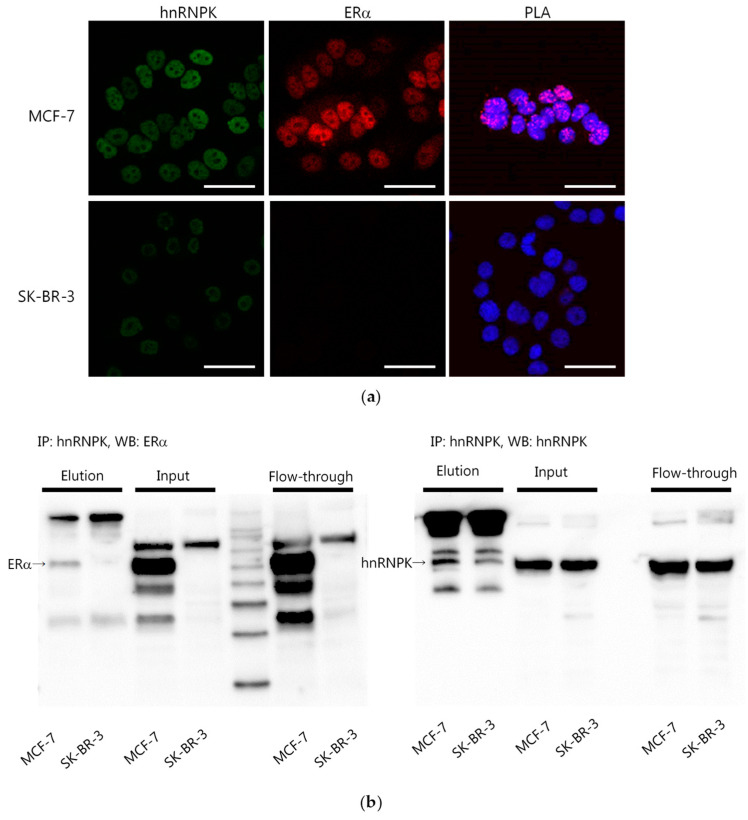
ERα and hnRNPK interaction. (**a**) Staining of hnRNPK and ERα using immunofluorescence (hnRNPK as green and ERα as red). Red dots (Texas red) indicate the interaction between hnRNPK and ERα detected using proximity ligation assay. Nuclei were stained blue (DAPI: 4′-6-diamidino-2-phenylindole). Scale bar, 100 µm. (**b**) MCF-7 and SK-BR-3 cells were immunoprecipitated with anti-hnRNPK antibodies. After SDS-PAGE, ERα (65 kDa) and hnRNPK (65 kDa) expressions were detected using Western blotting analysis (WB). Different materials were used in Western blots: MCF-7 and SK-BR-3 cells (Input), unbound material (Flow-through), and eluted protein fraction (Elution).

Since hnRNPK interacts with several other proteins [12], we explored the interaction between hnRNPK and ERα. First, we examined both hnRNPK and ERα expressions in MCF-7 and SK-BR-3 cells. Immunofluorescence results revealed that MCF-7 cells exhibited high expressions of both hnRNPK and ERα (Figure 3a). However, SK-BR-3 cells were hnRNPK-positive and ERα-negative (Figure 3a). The hnRNPK–ERα interaction was detected only in MCF-7 cells, but not in SK-BR-3 cells, using proximity ligation assay (PLA) (Figure 3a). In addition, the hnRNPK–ERα interaction was analyzed using immunoprecipitation (IP) (Figure 3b).

### 2.4. hnRNPK and ERα and Its Association with Clinicopathological Parameters of Patients with Breast Cancer

**Figure 4:**
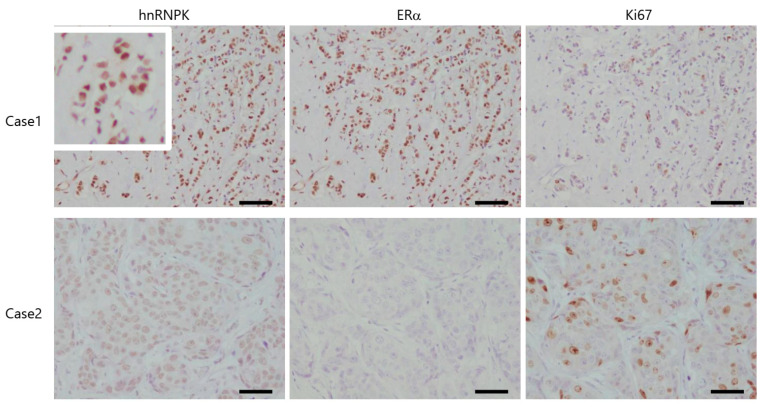
hnRNPK, ERα, and Ki67 immunoreactivity in patients with breast cancer. Case 1 yielded high hnRNPK and ERα expressions, but low Ki67 expression. Case 2 yielded low hnRNPK and ERα expressions, but high Ki67 expression. Scale bar, 50 µm.

Immunoreactivity of hnRNPK, ERα, and Ki67 was detected in the nuclei and counted in more than 1000 breast carcinoma cells. A labeling index (LI in %) was used to estimate the proportion or ratio of their immunoreactivity (Figure 4). Those with less than the median value (48.4%) were tentatively considered hnRNPK-negative, as previously reported [15].

hnRNPK expression was significantly higher in cases with low-stage, low pathologic T factor (pT), lymph node metastasis-negative cancers (Table 1). In addition, the status of hnRNPK immunoreactivity was significantly associated with high ERα and low Ki67 expressions (Table 1).

## 3. Discussion

In this study, E2 treatment significantly increased hnRNPK; the combination of E2 and ICI 182,780 decreased the expression of hnRNPK. In addition, hnRNPK knockdown using siRNAs resulted in decreased expression of ERα target gene pS2, and the depletion of hnRNPK decreased ERα expression. These results clearly indicated the involvement of hnRNPK in the ER-mediated signaling pathway. In addition, we examined the interaction between hnRNPK and ERα because hnRNPK interacts with several other proteins [12]. Using both PLA and IP analyses, the interaction between ERα and hnRNPK was detected in ERα-positive MCF-7 cells, but not in ERα-negative SK-BR-3 cells. Therefore, hnRNPK directly interacted with ERα and could function in patients with estrogen-dependent breast cancer. In addition, hnRNPK directly interacts with β-catenin, resulting in the stabilization and transactivation of β-catenin, which promotes the growth, invasion, and metastasis of neuroblastoma cells [14]. In addition, hnRNPK regulates and directly interacts with the androgen receptor translational apparatus in prostate cancer [16]. ERα protein stability is facilitated by several molecular mechanisms. For instance, in the cytoplasm, the retinoblastoma (RB) protein, a tumor suppressor, interacts with ERα and subsequently stabilizes ERα from degradation in breast carcinoma cells [17]. The interaction between RB and ERα allows the assembly of an intermediate complex with HSP90 in the cytoplasm [17]. In addition, HSP90 interacts with unliganded ERα and subsequently regulates its activity [18]. Therefore, the results of this study indicated that hnRNPK contributes to stabilizing ERα in the nucleus.

This is the first study to demonstrate the immunoreactivity of hnRNPK in breast cancer. Of particular note, hnRNPK expression was significantly higher in patients with low-stage, low-pT, lymph node metastasis-negative cancers. In this study, the immunoreactivity of hnRNPK was detected in the nucleus of breast cancer cells. However, Matta et al. have reported that hnRNPK was detected in both the nucleus and cytoplasm of head-and-neck/oral SCCA cells [5]. In addition, they have reported that the increased cytoplasmic expression in tumor cells suggested that nuclear–cytoplasmic translocation plays a pivotal role in the malignant transformation of oral SCCA [5]. Furthermore, our results demonstrated that hnRNPK was mainly expressed in the nuclei of breast carcinoma cells, indicating that hnRNPK functions as a tumor suppressor in breast carcinoma. Meanwhile, Chen et al. have reported that hnRNPK was mainly expressed in the nuclei of urinary bladder carcinoma cells, and a higher expression of nuclear hnRNPK was associated with a poor prognosis and served as an independent predictor of overall survival [7]. In patients with colon cancer, significant increases in both nuclear and cytoplasmic hnRNPK were observed among those with Dukes’ C stage [8]. Overall, patients with a low or negative nuclear hnRNPK score had poorer survival than those with a high nuclear hnRNPK score [8]. Carpenter et al. have reported that patients with p53 and hnRNPK expressions had worse clinical outcomes than those who did not express these two factors. hnRNPK could interact with various factors and exert different functions, depending on which factors it interacts with. In this study, we detected the interaction between hnRNPK and ERα in MCF-7 cells. In addition, the results of immunohistochemistry analysis indicated that hnRNPK expression was significantly higher in cases with high ERα and low Ki67 LI. ERα expression levels are positively associated with well-differentiated breast tumors and negatively associated with Ki67 LI [19]. Therefore, the interactions between hnRNPK and ERα could be involved in the suppression or inhibition of breast carcinoma cell proliferation.

In addition, ERα stability leads to a novel therapeutic approach for overcoming hormonal resistance in patients with luminal-type breast cancer [20]. In particular, hnRNPK and ERα interactions could result in the stabilization of ERα and enhance the therapeutic response of the patients to endocrine therapy; however, further investigations are required to clarify these findings.

## 4. Materials and Methods

### 4.1. Cell Culture

Both MCF-7 and SK-BR-3 cells were commercially obtained from the American Type Culture Collection (Manassas, VA, USA). They were maintained in a RPMI-1640 medium (Sigma-Aldrich, St. Louis, MO, USA) supplemented with 10% fetal bovine serum (FBS) (Biosera, Nuaille, France) and 100 µg/mL penicillin/streptomycin (Invitrogen, Carlsbad, CA, USA).

### 4.2. Breast Cancer Tissues

During surgery, breast cancer tissues were collected from 74 patients (age, 31–86 years). The specimens were fixed in 10% neutral formaldehyde and embedded in paraffin. Serial 3-µm tissue sections were used for immunostaining. We obtained approval for this study from the institutional review board of Tohoku University and Tohoku Kosai Hospital.

### 4.3. The Effects of E2 in MCF-7 Cells

First, MCF-7 cells were cultured in phenol-red-free RPMI-1640 (Sigma-Aldrich) supplemented with 10% dextran-coated charcoal-treated FBS for 48 h for estrogen-free experiments and then seeded at a density of 5.0 × 10^4^/mL into 6-well plates or a 60-mm dish. After 24 h, cells were treated with E2 (Wako Pure Chemical Industries, Osaka, Japan) or ICI 182,780 (Tocris Cookson, Ellisville, MO, USA).

### 4.4. The Effects of E2 in hnRNPK-Knockdown-MCF-7 Cells

MCF-7 cells were cultured in 6-well plates at a density of 1 × 10^5^ cells/mL. After 24 h, the cells were transfected with 5-nM hnRNPK-specific siRNAs (Sigma-Aldrich) or negative control siRNA (siCTL) (Ambion, Austin, TX, USA), by using Lipofectamine RNAiMAX (Invitrogen, Carlsbad, CA, USA), according to the manufacturer’s instructions. Cells were then treated with E2 for 24 h after transfection with the indicated siRNAs for 72 h.

### 4.5. Quantitative Reverse Transcription Real-Time PCR

Total RNA was extracted using TRI Reagent (Molecular Research Center, Cincinnati, OH, USA), cDNA was synthesized using a QuantiTect reverse transcription kit (Qiagen, Hilden, Germany). Real-time PCR was performed using the LightCycler 96 and FastStart Essential DNA Green Master (Roche, Basel, Switzerland). The PCR primer sequences were described in our previous studies [21]. RPL13A was used as a housekeeping gene.

### 4.6. Western Blotting

Western blotting was performed, as previously reported [22]. Proteins were electrophoresed on SDS-PAGE and transferred onto polyvinylidene fluoride membranes (Bio-Rad, Hercules, CA, USA). After blocking nonspecific sites, the membranes were incubated with rabbit monoclonal anti-hnRNPK antibody (1:5000, GTX61456; GeneTex, Irvine, CA, USA), rabbit monoclonal anti-pS2 antibody (1:1000, #15571; Cell Signaling Technology, Danvers, MA, USA), rabbit polyclonal anti-ERα antibody (1:500, sc-543; Santa Cruz Biotechnology, Dallas, TX, USA), or mouse monoclonal anti-β-actin antibody (1:1000, A5441, Sigma-Aldrich) and allowed to react with a secondary antibody. Protein bands were visualized using ECL Prime reagent (GE Healthcare, Chalfont St. Giles, UK).

### 4.7. Immunofluorescence

Immunofluorescence was performed, as previously reported [23]. MCF-7 or SK-BR-3 cells were seeded on EZ slides and cultured for 24 h. The cells were then fixed, permeabilized, and incubated with rabbit monoclonal anti-hnRNPK antibody (1:000) and mouse monoclonal anti-ERα antibody (1:50, NCL-ER-6F11; Leica Biosystems, Buffalo Grove, IL, USA) and then incubated with fluorescence-labeled secondary antibodies (1:500, Alexa Fluor 488 anti-rabbit and 1:500, Alexa Fluor 594 anti-mouse; Invitrogen). Then, the reacted slides were mounted with a mounting medium with DAPI (4′-6-diamidino-2-phenylindole).

### 4.8. In Situ PLA

Protein–protein interactions were detected using in situ PLA, as reported in our previous study [21,24]. The Duolink in situ PLA kit from Olink Bioscience (Olink Bioscience, Uppsala, Sweden) was used to detect hnRNPK and ERα interactions. Cells were fixed in 4% paraformaldehyde and subsequently incubated with a blocking solution, followed by overnight incubation with primary antibodies (1:1000, rabbit monoclonal anti-hnRNPK antibody and 1:50, mouse monoclonal anti-ERα antibody). Then, the cells were incubated with PLA PLUS and MINUS probes for mouse and rabbit and incubated with a ligation-ligase solution, followed by an amplification polymerase solution, according to the manufacturer’s instructions.

### 4.9. Immunoprecipitation

Immunoprecipitation was performed, as previously described, using a Dynabeads Protein G Immuno Precipitation Kit (Life Technologies, Gaithersburg, MD, USA) [24]. Dynabeads and Protein G were incubated with rabbit monoclonal anti-hnRNPK antibody. MCF-7- or SK-BR-3-derived protein lysates were incubated with the Dynabeads–anti-hnRNPK antibody complex. Then, ERα expression was examined with SDS-PAGE using the Dynabeads–anti-hnRNPK antibody–hnRNPK antigen complexes.

### 4.10. Immunohistochemistry

Immunohistochemical analysis was performed, as previously reported [22], using the biotin–streptavidin method with a Histofine kit (Nichirei Biosciences, Tokyo, Japan). Paraffin-embedded tissue sections were mounted on slides and heated in an autoclave at 121 °C for 5 min in a citrate buffer (pH 6.0). After blocking nonspecific sites, sections were incubated with rabbit monoclonal anti-hnRNPK antibody (1:300, GeneTex), mouse monoclonal anti-ERα antibody (1:50, Leica Biosystems), or mouse monoclonal anti-Ki67 antibody (1:100, M7240, Dako, Carpinteria, CA, USA). Stained sections were visualized using 3,3′-diaminobenzidine and counterstained with hematoxylin.

### 4.11. Statistical Analysis

Statistical analysis was performed using JMP 14 (SAS Institute Japan, Tokyo, Japan). *p* values less than 0.05 were used to denote statistical significance.

## 5. Conclusions

hnRNPK directly interacted with ERα and was involved in the ER-mediated signaling pathway in breast cancer. Furthermore, hnRNPK could be a novel target of endocrine therapy.

## Figures and Tables

**Table 1:** Association of hnRNPK with clinicopathological parameters in breast cancer patients.

		hnRNPK		
		Positive (*n* = 37)	Negative (*n* = 37)	*p* Value
Histological Grade	1	9	5	*p* = 0.0536
	2	19	14	
	3	8	18	
Stage	1	14	4	*p* = 0.0232
	2	14	21	
	3	7	9	
Pathologic T factor	1	20	9	*p* = 0.0092
	≥2	15	25	
Lymph node metastasis	Positive	12	22	*p* = 0.0175
	Negative	24	14	
ERα LI Median (Range)		72 (0–98)	18 (0–95)	*p* = 0.0027
PR LI Median (Range)		13 (0–83)	8 (0–90)	*p* = 0.7136
HER2	Positive	8	6	*p* = 0.5522
	Negative	29	31	
Ki67 LI Median (Range)		12 (0–31)	19 (3–53)	*p* = 0.0057

ER, estrogen receptor; PR, progesterone receptor; HER2, human epidermal growth factor receptor 2; LI, labeling index.

## Data Availability

The data that support the findings of this study are available from the corresponding author upon reasonable request.

## Supplementary Materials

The following are available online at https://www.mdpi.com/1422-0067/22/5/2581/s1.

## Abbreviations

E2	Estradiol
ER	Estrogen receptor
HER2	Human epidermal growth factor receptor 2
hnRNPK	Heterogeneous nuclear ribonucleoprotein
LI	Labeling index
SERM	Selective estrogen receptor modulator
siRNA	Small interfering RNA
PLA	Proximity ligation assay
PR	Progesterone receptor
