# Supplementary material for: Heterogeneous Nuclear Ribonucleoprotein K Is Involved in the Estrogen-Signaling Pathway in Breast Cancer
Source: Int J Mol Sci. 2021 Mar 4;22(5):2581. doi: 10.3390/ijms22052581 (PMC7962001; doi:10.3390/ijms22052581)
Supplement: Supplementary file 1 [file ijms-22-02581-s001.pdf]

Supplementary Figures

Figure S1.

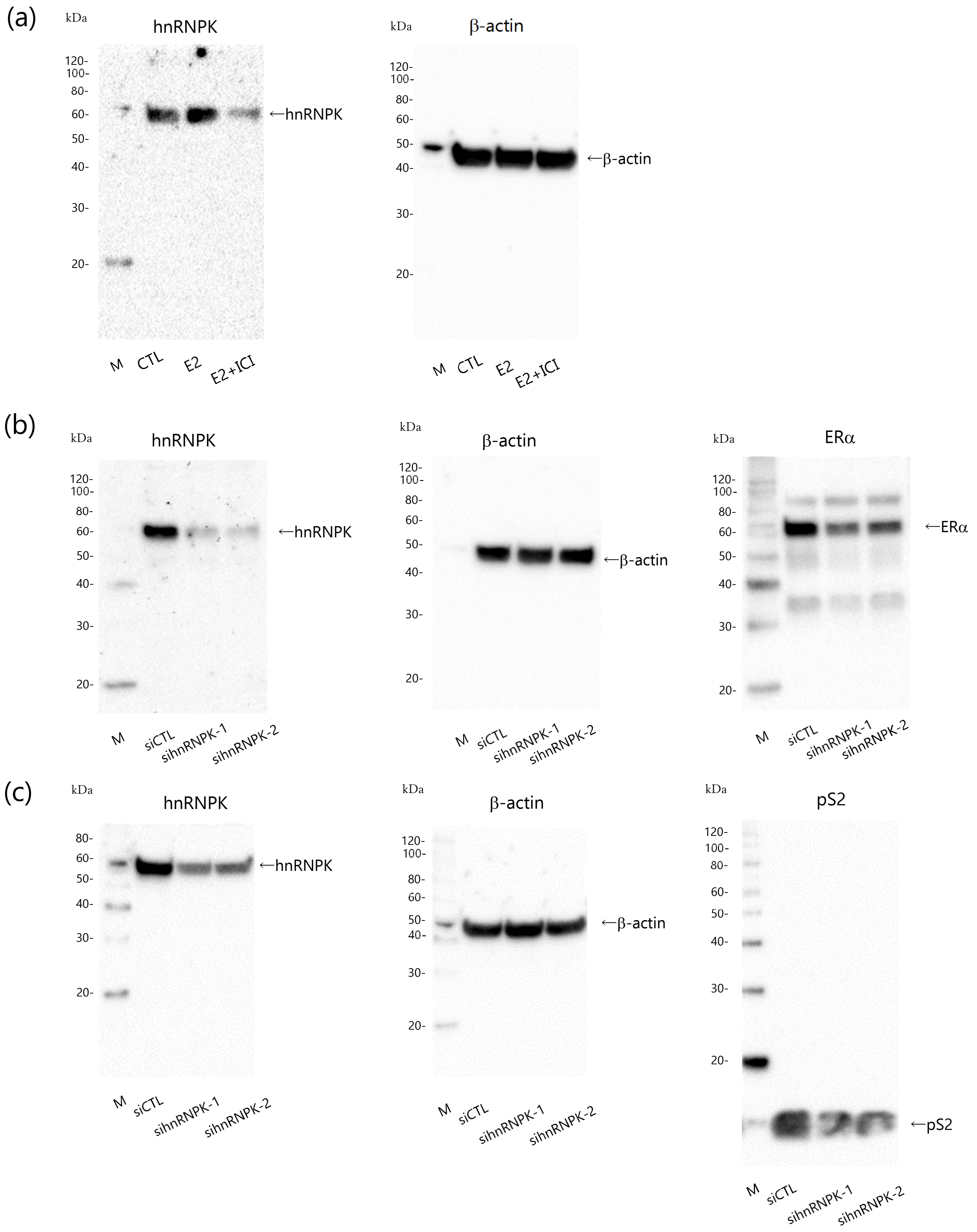

Figure S1. Full-length Western blot images (a) Full-length blot image for figure 1b. (b) Full-length blot image for figure 2a. (c) Full-length blot image for figure 2c. M, molecular weight marker.
